# Terlipressin Versus Norepinephrine for Septic Shock: A Systematic Review and Meta-Analysis

**DOI:** 10.3389/fphar.2019.01492

**Published:** 2019-12-23

**Authors:** Po Huang, Yuhong Guo, Bo Li, Qingquan Liu

**Affiliations:** ^1^ Beijing Hospital of Traditional Chinese Medicine, Capital Medical University, Beijing, China; ^2^ Infection and Immunity Laboratory, Beijing Institute of Traditional Chinese Medicine, Beijing, China; ^3^ Infection and Immunity Laboratory, Beijing Key Laboratory of Basic Research With Traditional Chinese Medicine on Infectious Diseases, Beijing, China

**Keywords:** terlipressin, norepinephrine, septic shock, meta-analysis, systematic review

## Abstract

**Purpose:** The meta-analysis aims to evaluate the efficacy and safety of terlipressin compared with norepinephrine for septic shock.

**Materials and Methods:** The relevant studies from MEDLINE, Cochrane Library, Embase were searched by two independent investigators. A variety of keywords were used to search the studies. Stata software (version 11.0, Stata Corp LP, College Station, TX, USA) was used for statistical analysis.

**Results:** A total of six studies were identified and incorporated into the meta-analysis. The results showed that there was no difference for 28-day mortality (RR = 0.99, 95% CI = [0.85,1.15], P = 0.849), AE (RR = 2.54, 95% CI = [0.58,11.08], P = 0.214), and MAP (SMD = -0.10, 95% CI = [-0.35,0.14], P = 0.405), OI, urinary output, Scr, total bilirubin, ALT, and AST between TP group and NE group. While TP could decrease HR at 24 and 48 h compared with NE.

**Conclusions:** Current results suggest that terlipressin showed no added survival benefit for septic shock when compared with norepinephrine, while terlipressin could decrease heart rate in the late phase of septic shock compared with norepinephrine without further liver and kidney injury.

**Systematic Review Registration:** PROSPERO (ID: CRD42019128743). Available online at: http://www.crd.york.ac.uk/PROSPERO/display_record.asp?ID=CRD42019128743.

## Introduction

Septic shock is a serious type of sepsis, mainly manifested as severe hypotension by low systolic pressure (≤90 mmHg) or mean arterial blood pressure (≤65 mmHg) accompanied by signs of hypoperfusion (Annane et al., 2005). The previous study found that septic shock is the fifth leading cause due to premature mortality (Murray et al., 2013). According to the latest guideline (Singer et al., 2016), standard rescue measures for septic shock include circulation, respiratory support, and antibiotic application. Among these, effective hemodynamic support and fluid resuscitation are the key measures for successful treatment of septic shock.

For septic shock, vasoactive drugs are important means to maintain the stability of hemodynamics and ensure the perfusion of major organs. Norepinephrine (NE) is the first-line drug for septic shock (Singer et al., 2016). However, NE mainly acts on the alpha-adrenergic receptor (alpha-receptor) of peripheral vascular resistance, which can increase cardiac after-load and thus reduce the volume responsiveness of patients. What’s more, NE may induce life-threatening arrhythmia (i.e., ventricular tachycardia, ventricular fibrillation) (Anantasit, 2014). Herein, alternative, non-adrenergic vasopressors are desirable as first- or second-line treatment of sepsis-associated vasodilation, hoping that they can play a synergistic effect of NE or reduce the dosage of NE, so as to minimize the occurrence of adverse reactions.

Recently, a number of randomized controlled trials (RCTs) compared terlipressin (TP) with NE in the treatment of septic shock, but there are still some controversies in terms of mortality and adverse reactions (Westphal et al., 2003; Albanese, 2005; Masarwa et al., 2017). According to the pharmacological research, TP has stronger binding specificity for the V1 receptor, which made it more effective than other vasoactive drugs in sepsis-induced refractory hypotension. What’s more, previous studies showed the mortality and doses of NE in patients with septic shock could be decreased by TP (Rehberg et al., 2009; Serpa Neto et al., 2012). While the exited two reviews had different results on vasoactive drugs for septic shock (Polito et al., 2012; Serpa Neto et al., 2012). In view of this, it is necessary to perform a meta-analysis to evaluate the efficacy and safety of TP and NE for septic shock, so as to provide evidence-based evidence for the treatment of septic shock.

## Methods

This meta-analysis was conducted according to the recommendations and checklist from the preferred reporting items for systematic review and meta-analysis (PRISMA) statement (Panic et al., 2013).

### Search Strategy

The relevant RCTs from Medline, Cochrane Library, and Embase were searched. The duration is from their inception to March 2019. Besides, references of the selected articles were also searched as supplementary search. The search strategy of Medline was shown in **Supplemental Figure S1**.

### Eligibility Criteria of Original Studies

Inclusion criteria: (1) Participants: Adult participants suffered from septic shock, the diagnostic criteria should be explicit and normative, regardless of the gender and ethnicity; (2) Intervention: TP; (3) Control: NE; (4) Outcomes: Primary outcome: ICU mortality, Secondary outcomes: Adverse events (AEs), mean arterial pressure (MAP), heart rate (HR), urinary output, serum creatinine (Scr), oxygenation index (OI), total bilirubin, alanine transaminase (ALT), aspartate aminotransferase (AST). (5) Study design: RCT.

Exclusion criteria: (1) TP plus dopamine or NE as the intervention; (2) duplicate publication and the research data cannot be achieved.

### Study Selection

Two reviewers independently identified studies through inclusion criteria by screening the title and abstract of each record and retrieved their full text if necessary. Any disagreement between the two reviewers was solved with a discussion with a third reviewer. Otherwise, the agreement was accomplished by a consensus.

### Data Extraction and Quality Assessment

Two reviewers independently extracted the information from the included studies. The information includes the first author, published year, sample size, the baseline of the included studies, intervention, and endpoints. Any disagreement between the two reviewers was solved with a discussion with a third reviewer. Otherwise, the agreement was accomplished by a consensus.

A modified Jadad scale was used to assess the quality of the selected RCTs. The items of modified Jadad scale were listed in the protocol (Jadad et al., 1996). According to its principle, 1–3 scores indicating a low-quality study and 4–7 scores indicating a high-quality study, the maximum of Jadad score is 7.

### Data Synthesis

Statistical analysis of this meta-analysis was performed by Stata software (version 11.0, Stata Corp LP, USA). Risk ratios (RRs) and 95% confidence intervals (CIs) were employed to assess the dichotomous endpoints. If the continuous variables need to be analyzed, we would choose the mean difference and its 95% CI. The heterogeneity was assessed by I^2^ test (low heterogeneity is defined as I^2^ ≤ 25%; if I^2^≥75%, it will be considered as high heterogeneity). The fixed-effect model was applied if there was no or low heterogeneity, and pooled RRs were estimated using the Mantel–Haenszel method. Publication bias was assessed if there are more than 10 studies in one outcome. All hypotheses were tested at the alpha = 0.05 level.

### Subgroups Analysis

Subgroups will be analyzed based on the different types of adverse events (AEs) or different time (MAP, HR, urinary output, Scr, OI, total bilirubin, ALT, AST).

## Results

### Description of Included Studies

**Figure 1:**
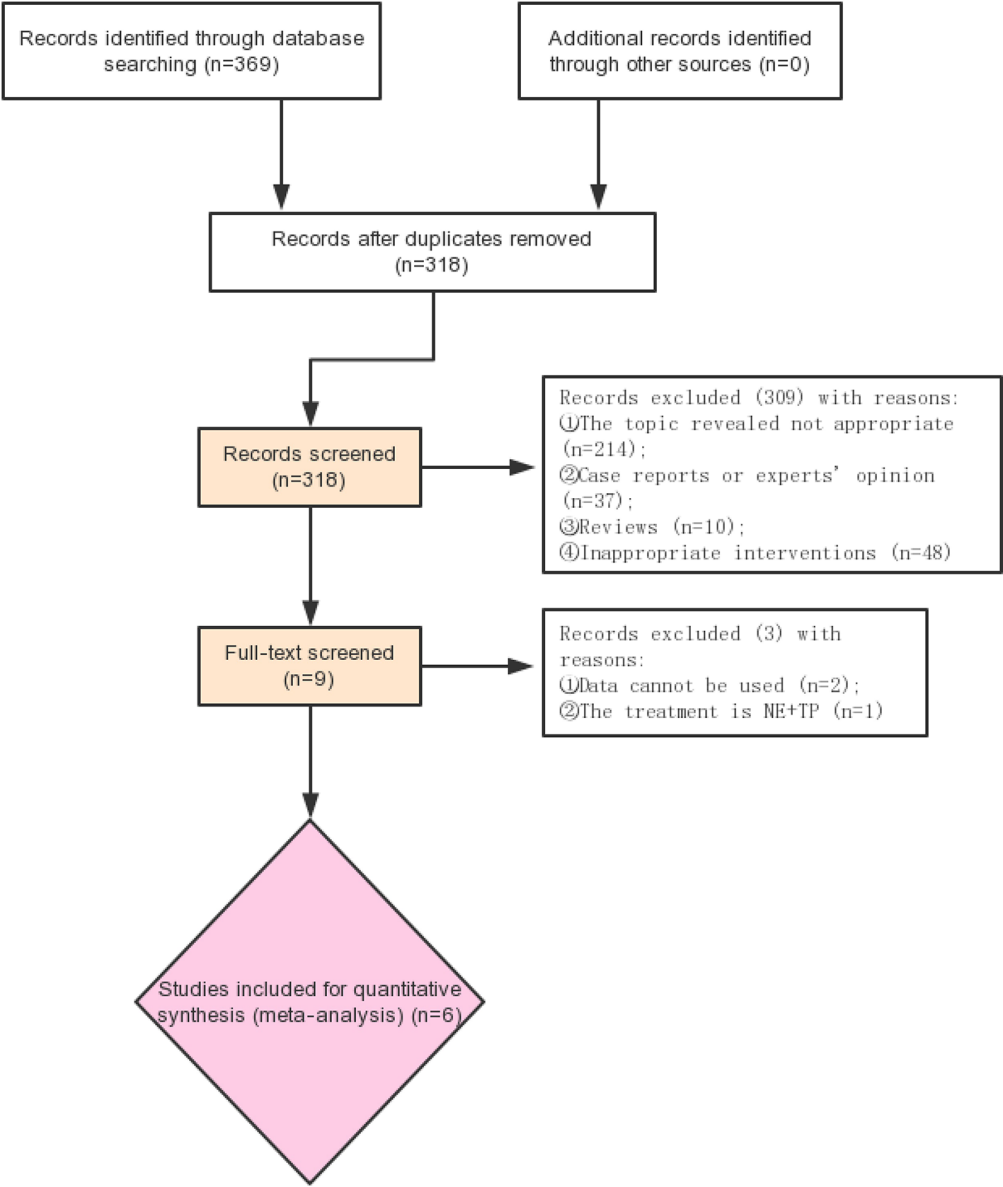
Flow chart of included studies selection.

Retrieval process of the included studies is shown in **Figure 1**. Six studies (Albanese, 2005; Morelli et al., 2008; Morelli et al., 2009; Liu et al., 2017; Choudhury et al., 2017; Liu, 2018) with 756 participants were included (**Figure 1**). The characteristics of the six studies are listed in **Table 1**. The quality of the above RCTs is shown in **Supplementary Table S1**.

**Table 1:** The characteristics of the included studies.

Study	No. of participants	Mean age (year)	Intervention		Outcomes
			Experimental group	Control group	
Albanese et al., 2005	N = 20 (T = 10; C = 10)	T: 66 (23–79) C: 65 (24–76)	Terlipressin (1 mg bolus)	NE (0.3 ug/kg/min)	ICU mortality, Urinary output
Chen et al., 2017	N = 57 (T = 31; C = 26)	T:58.5 ± 17.8 C:55.7 ± 16.1	Terlipressin (0.01–0.04 U/min)	NE(> 1 ug/min)	ICU mortality, MAP, HR, OI, Urinary output, Scr
Choudhury et al., 2016	N = 84 (T = 42; C = 42)	T:46.76 ± 12.11 C: 48.29 ± 12.53	Terlipressin (1.3–5.2 ug/min)	NE (7.5 ug/min)	ICU mortality, adverse events
Liu et al., 2018	N = 526 (T = 260; C = 266)	T: 60.93 ± 15.86 C:61.09 ± 16.20	Terlipressin (20–160 ug/h with maximum infusion rate of 4 mg/day)	NE (4–30 ug/min)	ICU mortality, adverse events
Morelli A et al., 2008	N = 39 (T = 19; C = 20)	T: 66 (28–84) C: 67(29–83)	Terlipressin (1 mg bolus)	NE (84 ug/min)	ICU mortality, urinary output, total bilirubin, ALT, AST
Morelli A et al., 2009	N = 30 (T = 15; C = 15)	T: 67 (60–71) C: 64 (59–72)	Terlipressin (1.3 ug/kg/h)	NE (15 ug/min)	ICU mortality, MAP, HR, OI, Urinary output, Scr, total bilirubin, ALT, AST

### Quality Assessment

Of the included studies, four of the six (66.7%) studies (Morelli et al., 2008; Morelli et al., 2009; Chen et al., 2017; Liu, 2018) obtained high scores (>3) assessed by the modified Jadad scale. Only one study (Liu, 2018) reported allocation concealment. Two of the six studies (Albanese, 2005; Choudhury et al., 2017) didn’t report blinding method. The details of the quality assessment could be seen in **Supplementary Table S1**.

### Primary Outcome

**Figure 2:**
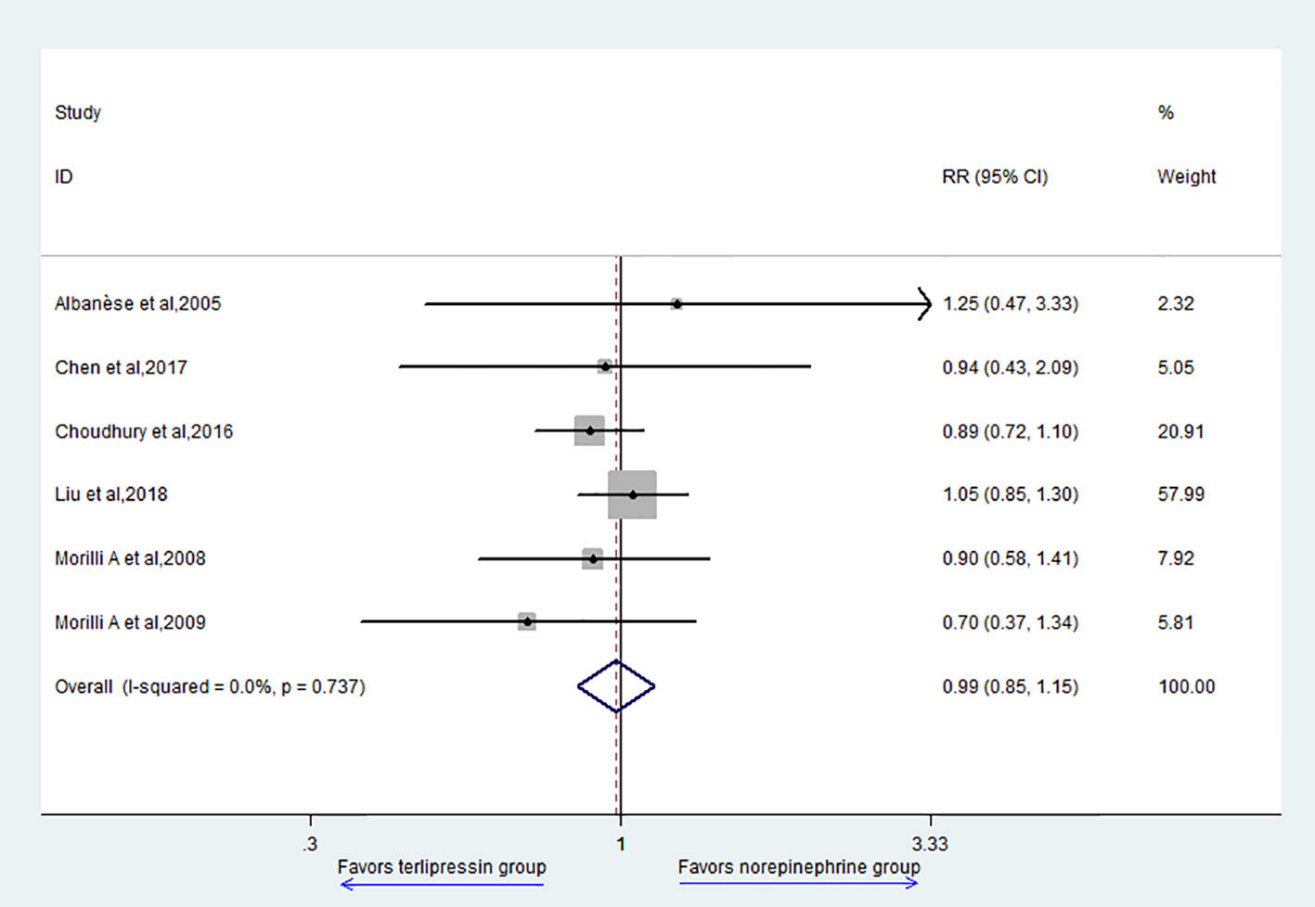
Forest plot of 28–day mortality.

The primary outcome of this study is 28-day mortality. Six studies reported 28-day mortality and the result showed that there was no difference between TP group and NE group (RR = 0.99, 95% CI = [0.85,1.15], P = 0.849) (**Figure 2**).

### Secondary Outcomes

**Figure 3:**
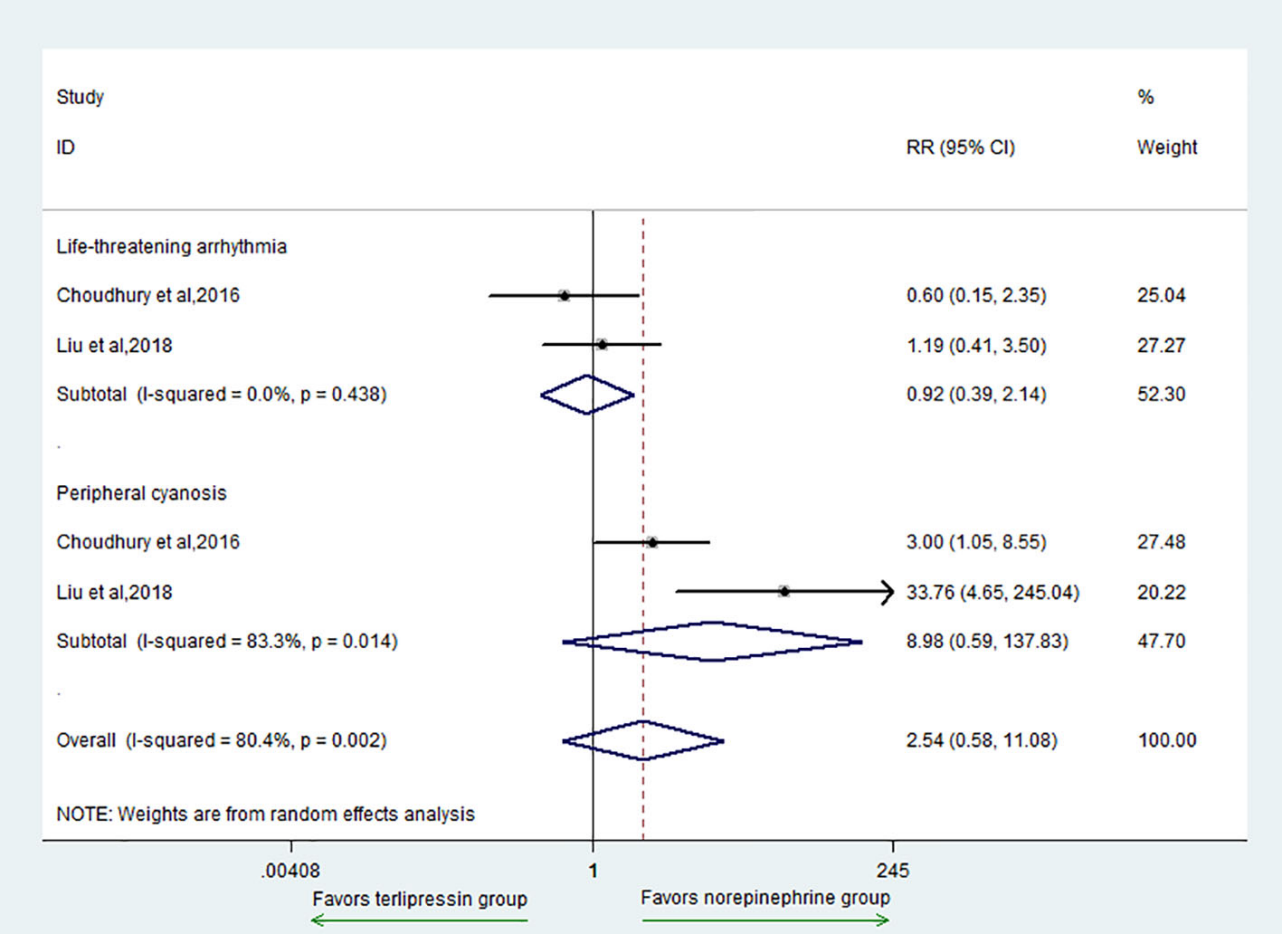
Forest plot of AE.

The secondary outcomes included AE, MAP, HR, urinary output, Scr, OI, and total bilirubin. For the outcome of AE, there was no difference between the two groups (RR = 2.54, 95% CI = [0.58, 11.08], P = 0.214) (**Figure 3**). In addition, for subgroup analysis, there was no difference in the adverse events of life-threatening arrhythmia (RR = 0.92, 95% CI = [0.39,2.14], P = 0.841) and peripheral cyanosis (RR = 8.98, 95% CI = [0.59,137.83], P = 0.115) between the two groups.

**Figure 4:**
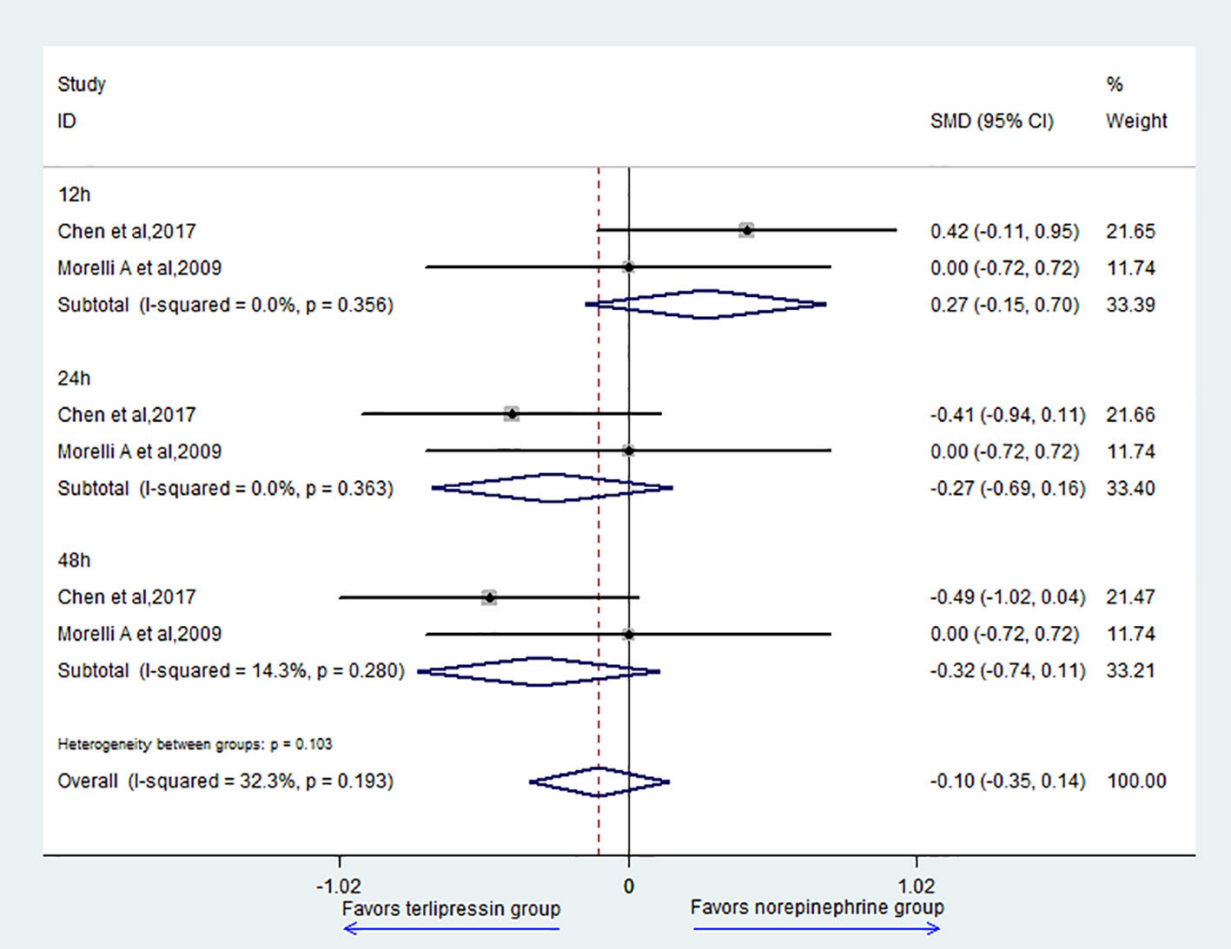
Forest plot of MAP.

Two studies (Morelli et al., 2009; Liu et al., 2017) reported the data of MAP; the result showed that there was no difference between the two groups (SMD = -0.10, 95% CI = [-0.35,0.14], P = 0.405) (**Figure 4**). For subgroup analysis, there was no difference in 12 h (SMD = 0.27, 95% CI = [-0.15,0.70], P = 0.21), 24 h (SMD = -0.27, 95% CI = [-0.69,0.16], P = 0.216), and 48 h (SMD = -0.32, 95% CI = [-0.74,0.11], P = 0.144) between the two groups.

**Figure 5:**
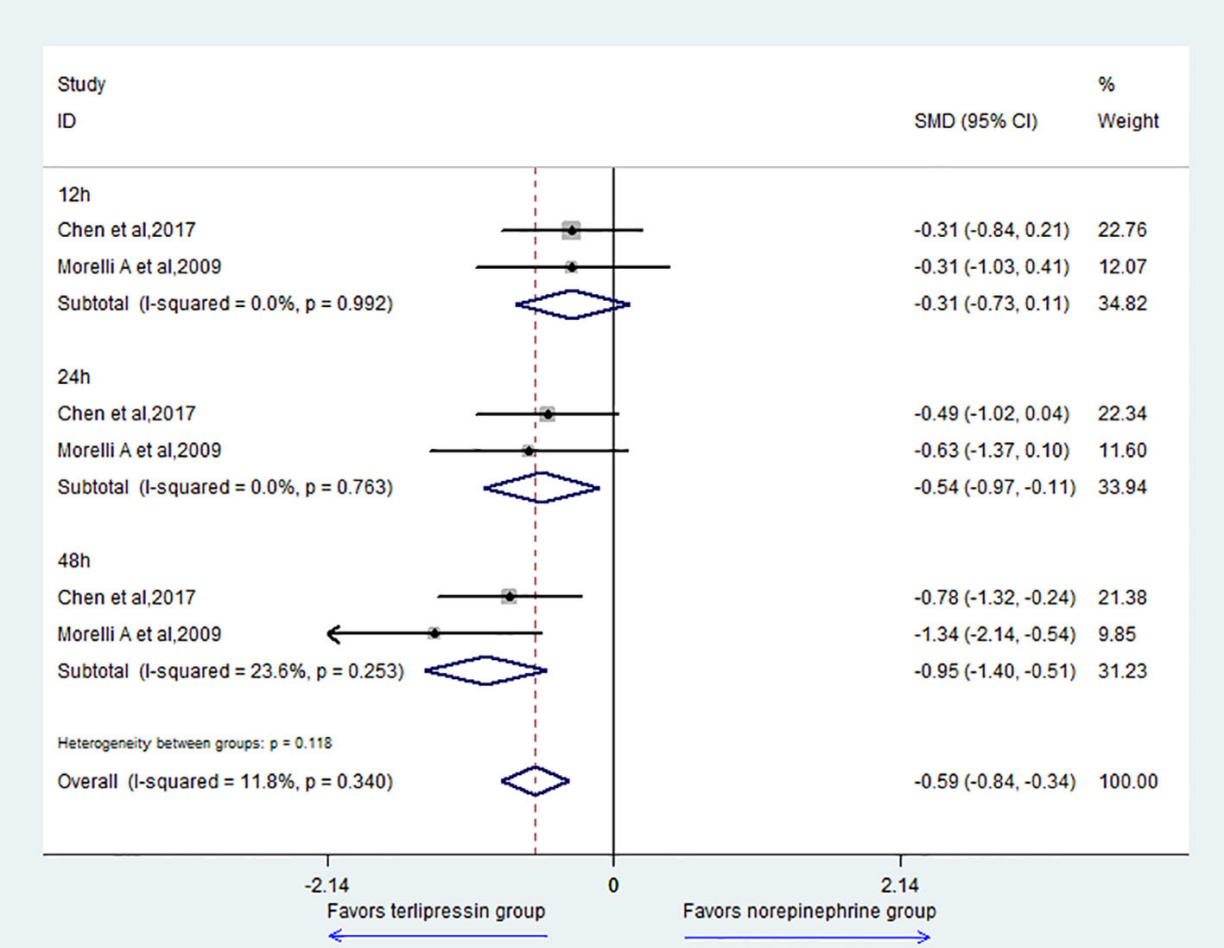
Forest plot of HR.

For the outcome of the HR, the result showed that the TP group could decrease HR compared with the NE group (SMD = -0.59, 95% CI = [-0.84,-0.34], P = 0.000) (**Figure 5**). For subgroup analysis, there was no difference at 12 h (SMD = -0.31, 95% CI = [-0.73,0.11], P = 0.151). However, the TP group could decrease HR at 24 h (SMD = -0.54, 95% CI = [-0.97,-0.11], P = 0.014) and 48 h (SMD = -0.95, 95% CI = [-1.40,-0.51], P = 0.000).

Two studies (Morelli et al., 2009; Liu et al., 2017) reported the data of OI; the result showed that there was no difference between the two groups (SMD = 0.00, 95% CI = [-0.24,0.25], P = 0.97) (**Supplemental Figure S2**). For subgroup analysis, there was no difference in 12 h (SMD = -0.04, 95% CI = [-0.47,0.38], P = 0.841), 24 h (SMD = 0.03, 95% CI = [-0.39,0.45], P = 0.882), and 48 h (SMD = 0.03, 95% CI = [-0.40,0.45], P = 0.907) between the two groups.

For outcome of urinary output, the result showed that there was no difference between the two groups (SMD = 0.14, 95% CI = [-0.12,0.39], P = 0.304) (**Supplemental Figure S3**). For subgroup analysis, there was no difference in 4 h (SMD = 0.02, 95% CI = [-1.14,1.18], P = 0.977), 12 h (SMD = 0.18, 95% CI = [-0.24,0.61], P = 0.394), 24 h (SMD = -0.02, 95% CI = [-0.44,0.41], P = 0.937), and 48 h (SMD = 0.21, 95% CI = [-0.58,0.99], P = 0.609) between the two groups.

Two studies (Morelli et al., 2009; Liu et al., 2017) reported the data of Scr; the result showed that there was no difference between the two groups (SMD = 0.11, 95% CI = [-0.19,0.41], P = 0.481) (**Supplemental Figure S4**). For subgroup analysis, there was no difference in 24 h (SMD = 0.23, 95% CI = [-0.19,0.65], P = 0.286) and 48 h (SMD = -0.02, 95% CI = [-0.44,0.41], P = 0.944) between the two groups.

For outcome of total bilirubin, the result showed that there was no difference between the two groups (SMD = -0.59, 95% CI = [-1.25,0.08], P = 0.084) (**Supplemental Figure S5**). For subgroup analysis, there was no difference in 12 h (SMD = -0.68, 95% CI = [-1.94,0.58], P = 0.288) and 24 h (SMD = -0.52, 95% CI = [-1.59,0.54], P = 0.339) between the two groups.

For outcome of ALT, the result showed that there was no difference between the two groups (SMD = -0.15, 95% CI = [-0.50,0.19], P = 0.383) (**Supplemental Figure S6**). For subgroup analysis, there was no difference in 12 h (SMD = -0.10, 95% CI = [-0.81,0.62], P = 0.789) and 24 h (SMD = -0.23, 95% CI = [-0.71,0.24], P = 0.333) between the two groups.

Two studies (Morelli et al., 2008; Morelli et al., 2009) reported the data of AST; the result showed that there was no difference between the two groups (SMD = -0.08, 95% CI = [-0.44,0.28], P = 0.657) (**Supplemental Figure S7**). For subgroup analysis, there was no difference in 12 h (SMD = -0.05, 95% CI = [-0.77,0.68], P = 0.901) and 24 h (SMD = -0.14, 95% CI = [-0.63,0.36], P = 0.586) between the two groups.

## Discussion

### Summary of Findings

To our knowledge, our study is the first meta-analysis to solely evaluate TP versus NE for septic shock patients. The results showed that there was no difference for 28-day mortality (RR = 0.99, 95% CI = [0.85,1.15], P = 0.849), AE (RR = 2.54, 95% CI = [0.58,11.08], P = 0.214), and MAP (SMD = -0.10, 95% CI = [-0.35,0.14], P = 0.405) between TP group and NE group. While TP could decrease HR at 24 and 48 h compared with NE (**Table 2**). Meanwhile, there was no difference for OI, urinary output, Scr, total bilirubin, ALT, and AST. That is to say, compared with NE, TP could decrease HR at 24 and 48 h without further liver and kidney injury, though TP showed no added survival benefit for septic shock.

**Table 2:** Summary of meta-analysis.

Outcomes	Subgroups	No. of studies	No. of participants	Effect size (95% CI)	I^2^	Z	P
ICU mortality	NA	6	756	RR, 0.99 [0.85, 1.15]	0	0.19	0.849
Adverse events	a	2	546	RR, 0.92 [0.39, 2.14]	0	0.20	0.841
	b	2	546	RR, 8.98 [0.59, 137.83]	83.3%	1.58	0.115
	Overall	2	546	RR, 2.54 [0.58, 11.08]	80.4%	1.24	0.214
MAP	12 h	2	87	SMD, 0.27 [-0.15, 0.70]	0	1.25	0.210
	24 h	2	87	SMD, -0.27 [-0.69, 0.16]	0	1.24	0.216
	48 h	2	87	SMD, -0.32 [-0.74, 0.11]	14.3%	1.46	0.144
	Overall	2	87	SMD, -0.10 [-0.35, 0.14]	32.3%	0.83	0.405
HR	12 h	2	87	SMD, -0.31 [-0.73, 0.11]	0	1.43	0.151
	24 h	2	87	SMD, -0.54 [-0.97, -0.11]	0	2.47	0.014
	48 h	2	87	SMD, -0.95 [-1.40, -0.51]	23.6%	4.18	0.000
	Overall	2	87	SMD, -0.59 [-0.84, -0.34]	11.8%	4.62	0.000
OI	12 h	2	87	SMD, -0.04 [-0.47,0.38]	18.8%	0.2	0.841
	24 h	2	87	SMD, 0.03 [-0.39,0.45]	0	0.15	0.882
	48 h	2	87	SMD, 0.03 [-0.40,0.45]	44.8%	0.12	0.907
	Overall	2	87	SMD, 0.00 [-0.24,0.25]	0	0.04	0.970
Urinary output	4 h	2	59	SMD, 0.02 [-1.14,1.18]	77.5%	0.03	0.977
	12 h	2	87	SMD, 0.18 [-0.24,0.61]	0	0.85	0.394
	24 h	2	87	SMD, -0.02 [-0.44,0.41]	0	0.08	0.937
	48 h	2	87	SMD, 0.21 [-0.58,0.99]	68.1%	0.51	0.609
	Overall	4	146	SMD, 0.14 [-0.12,0.39]	24.4%	1.03	0.304
Scr	24 h	2	87	SMD, 0.23 [-0.19,0.65]	0	1.07	0.286
	48 h	2	87	SMD, -0.02 [-0.44,0.41]	0	0.07	0.944
	Overall	2	87	SMD, 0.11 [-0.19,0.41]	0	0.70	0.481
Total bilirubin	12 h	2	59	SMD, -0.68 [-1.94,0.58]	83.7%	1.08	0.288
	24 h	2	59	SMD, -0.52 [-1.59,0.54]	78.3%	0.93	0.339
	Overall	2	59	SMD, -0.59 [-1.25,0.08]	72.4%	2.17	0.084
ALT	12 h	2	59	SMD, -0.10 [-0.81,0.62]	54.9%	0.33	0.789
	24 h	2	59	SMD, -0.23 [-0.71,0.24]	0	0.97	0.333
	Overall	2	59	SMD, -0.15 [-0.50,0.19]	6.1%	0.89	0.383
AST	12 h	2	59	SMD, -0.05 [-0.77,0.68]	55.9%	0.09	0.901
	24 h	2	59	SMD, -0.14 [-0.63,0.36]	7.4%	0.67	0.586
	Overall	2	59	SMD, -0.08 [-0.44,0.28]	13.4%	0.59	0.657

MAP, mean arterial pressure; HR, heart rate; NA, not available; a, Life-threatening arrhythmia; b, Peripheral cyanosis; OI, Oxygenation index; Scr, Serum creatinine; ALT, Alanine aminotransferase; AST, Aspartate transaminase; CI, confidence interval; RR, relative risks; WMD, weighted mean difference.

### The Significance of This Study

For decades ago, the application of NE and dopamine in septic shock has been controversial, and there were numerous studies and reviews to evaluate the efficacy and safety of these agents for septic shock (Annane et al., 2007; Patel et al., 2010; De Backer et al., 2012; Vasu Tajender et al., 2012). Based on this evidence, the guideline made a specific recommendation that NE should be used as a first-line drug for septic shock and dopamine is not recommended (Singer et al., 2016). However, we cannot ignore the clinical side effects of catecholamine resistance (Yildizdas et al., 2008) and NE, such as severe malignant arrhythmias (Gamper et al., 2016). Hence, seeking for alternative vasoactive drugs (especially for TP) has become the focus of septic shock treatment and research.

Recently, accumulating evidence suggested that TP has satisfactory effect to stabilize cardiocirculatory functions both in experimental and clinical sepsis (Westphal et al., 2003). More importantly, TP could be used in patients with catecholamine-refractory septic shock who are resistant to catecholamines (Leone et al., 2004; Yildizdas et al., 2008). Thus, TP seems to be an effective rescue strategy for patients who suffered from catecholamine-resistant septic shock (O’Brien et al., 2002). However, TP may be associated with unfavorable effects, such as impairment in cardiac index (Albanese, 2005), ischemic skin lesions (Dünser et al., 2003), and systemic oxygen delivery (Dünser et al., 2001).

### The Pharmacological Mechanism of TP

Previous study investigated that activation of the V1 receptor results in vasoconstriction and arterial blood pressure ascent (Lange et al., 2008). However, arginine-vasopressin (AVP) has no selectivity for the V1 receptor and may produce side effects due to activation of the other receptors (Torgersen et al., 2010; Salazar et al., 2015). TP has a much stronger selectivity to the V1 receptor than to other receptors. The chemical structure of TP is shown in **Supplemental Figure S8**.

### Strengths and Limitations

Our study is the first to directly compare TP with NE for septic shock, and a number of endpoints including ICU mortality, adverse events, heart rate, and mean arterial pressure were evaluated. Meanwhile, subgroups were conducted based on the different types of adverse events (AEs) or different time (MAP and HR). However, there were some limitations. First is due to the small sample size that limited the precision of the overall review. Second, we didn’t conduct a subgroup meta-analysis based on the different timing of TP usage, since there were only some studies performed on animals (Asfar et al., 2005; Kampmeier et al., 2018) and case series (Zeballos et al., 2006; Morelli et al., 2007).

### The Significance of This Study for Future Clinical Practice and Research

Morelli et al. (Morelli et al., 2007) conducted a case series; the results showed that skin necrosis occurred in patients treated with NE as first-line vasopressor and a delayed TP infusion. However, the patients treated with TP as first-line vasopressor survived and showed no signs of TP-related AEs. Taken together, with results from experimental studies (Asfar et al., 2005; Lange et al., 2007) and previous clinical case reports (Jolley et al., 2003; Zeballos et al., 2006), early low-dose infusion of TP may potentially be superior to a later resort strategy in septic shock. Recently, Xiao et al. (2016) reported that low dose of TP continuous infusion combined with NE has synergistic effect in septic shock patients. Taken together with the results from our meta-analysis, low dose of TP may be recommended as the first-line vasopressor for refractory hypotension. Thus, further studies are needed to evaluate the efficacy and safety of low dose of TP for refractory hypotension after septic shock.

## Conclusions

Current results suggest that TP showed no added survival benefit for septic shock when compared with NE. While TP could decrease heart rate in the late phase of septic shock compared with NE without further liver and kidney injury.

## Data Availability Statement

The datasets analyzed in this article are not publicly available. Requests to access the datasets should be directed to liuqingquan_2003@126.com.

## Author Contributions

PH participated in the sequence alignment and the design of the study, drafted the manuscript and performed the statistical analysis. YG carried out the analysis. BL participated in the sequence alignment. QL conceived of the study, participated in its design and coordination and helped to draft the manuscript. All authors read and approved the final manuscript.

## Conflict of Interest

The authors declare that the research was conducted in the absence of any commercial or financial relationships that could be construed as a potential conflict of interest.

## Abbreviations

NE, norepinephrine; TP, terlipressin; RCTs, randomized controlled trials; AEs, adverse events; RR, risk ratio; CIs, confidence intervals; SMD, standard weight deviations; MAP, mean arterial pressure; HR, heart rate; AVP, arginine-vasopressin.

## Supplementary Material

Figure S1 The search strategy of Medline.

Figure S2 Forest plot of oxygenation index.

Figure S3 Forest plot of urinary output.

Figure S4 Forest plot of Scr.

Figure S5 Forest plot of total bilirubin.

Figure S6 Forest plot of ALT.

Figure S7 Forest plot of AST.

Figure S8 The chemical structure of TP.

Table S1 The details of the quality assessment.
