# Supplementary figures and images for: Terlipressin Versus Norepinephrine for Septic Shock: A Systematic Review and Meta-Analysis
Source: Front Pharmacol. 2019 Dec 23;10:1492. doi: 10.3389/fphar.2019.01492 (PMC6936170; doi:10.3389/fphar.2019.01492)

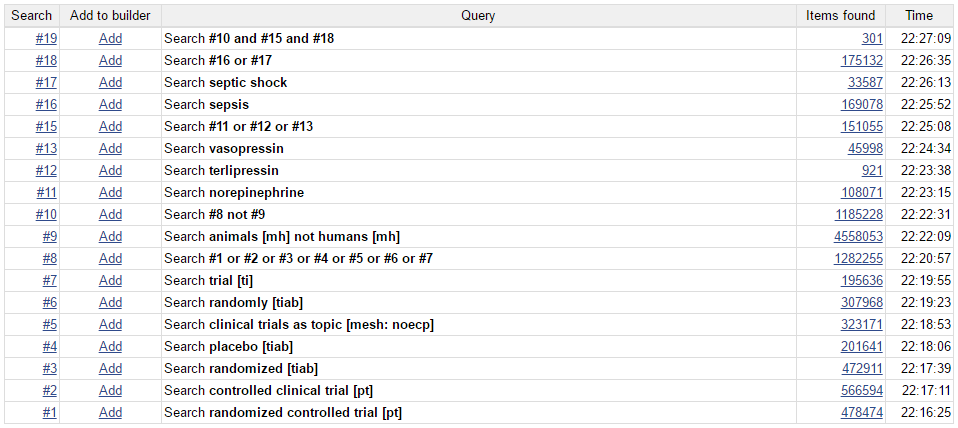

Supplement: Figure S1 — The search strategy of Medline. [file Image_1.png]

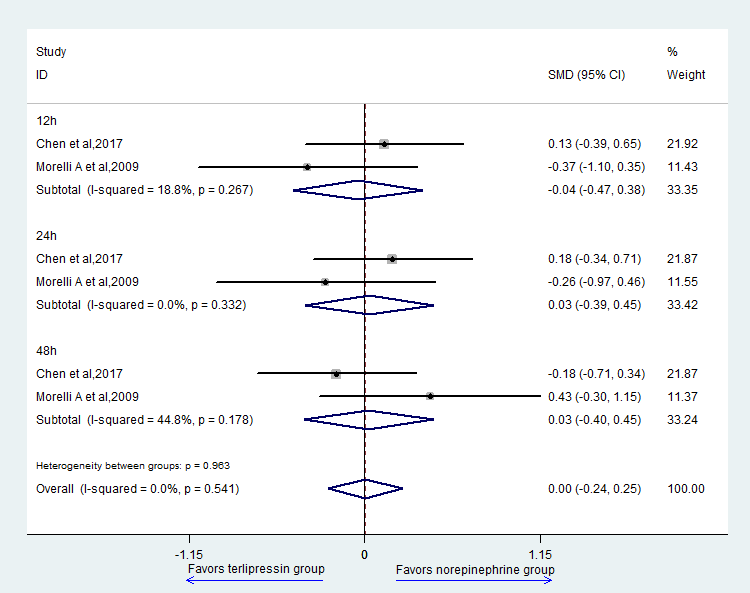

Supplement: Figure S2 — Forest plot of oxygenation index. [file Image_2.tif]

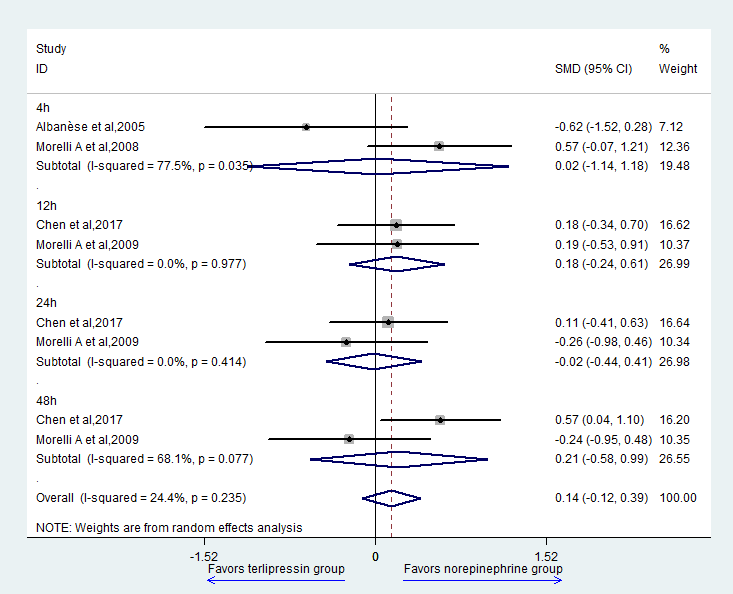

Supplement: Figure S3 — Forest plot of urinary output. [file Image_3.tif]

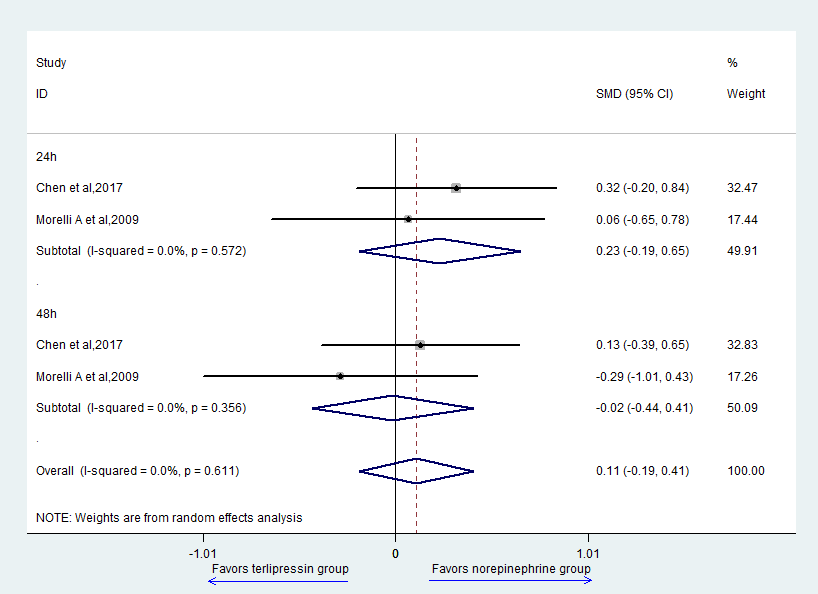

Supplement: Figure S4 — Forest plot of Scr. [file Image_4.tif]

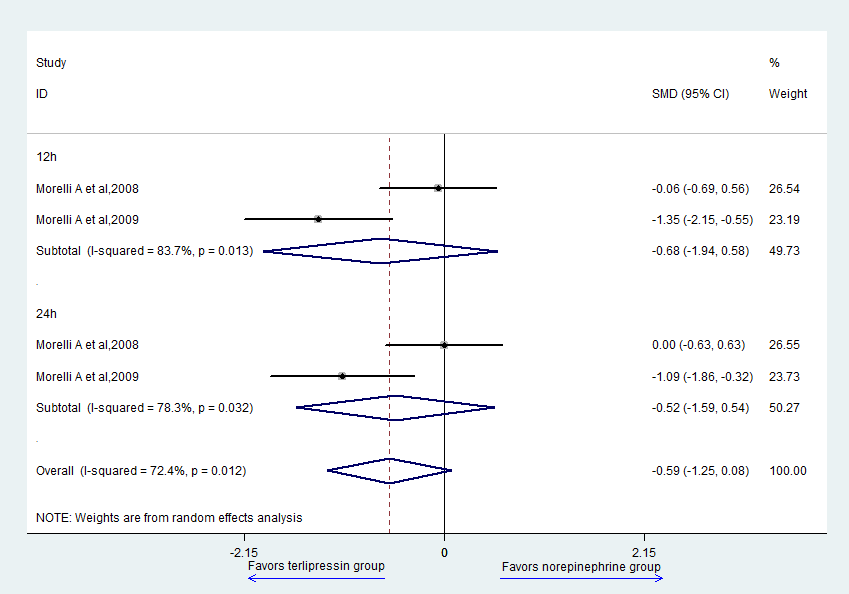

Supplement: Figure S5 — Forest plot of total bilirubin. [file Image_5.tif]

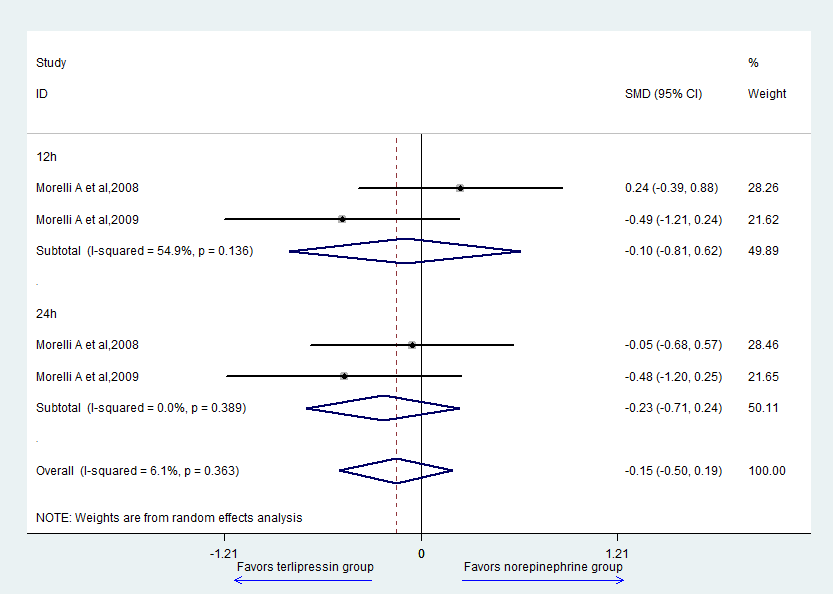

Supplement: Figure S6 — Forest plot of ALT. [file Image_6.tif]

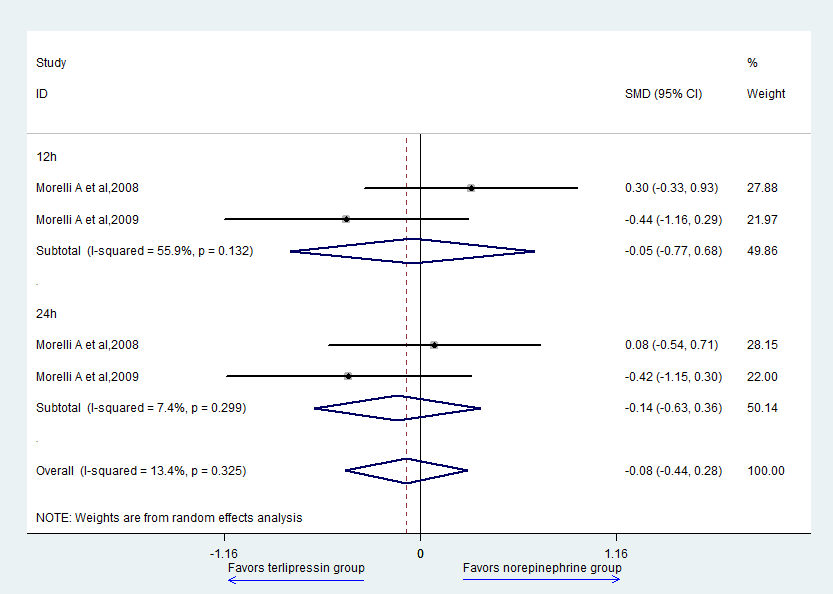

Supplement: Figure S7 — Forest plot of AST. [file Image_7.tif]

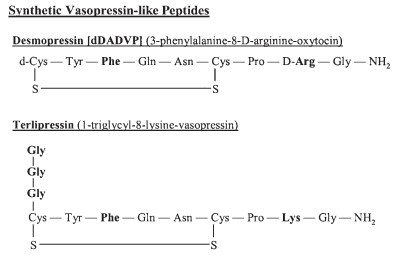

Supplement: Figure S8 — The chemical structure of TP. [file Image_8.jpeg]
