# Supplementary material for: Terlipressin Versus Norepinephrine for Septic Shock: A Systematic Review and Meta-Analysis
Source: Front Pharmacol. 2019 Dec 23;10:1492. doi: 10.3389/fphar.2019.01492 (PMC6936170; doi:10.3389/fphar.2019.01492)
Supplement: Table S1 — The details of the quality assessment. [file Table_1.pdf]

Table S1 The quality of included studies

Study	The generation of random sequences	Allocation concealment	Blinding method	Reasons for withdrawal and dropout	Total scores
Albanèse et al,2005	2	0	0	1	3
Chen et al,2017	2	0	2	1	5
Choudhury et al,2016	2	0	0	1	3
Liu et al,2018	2	2	2	1	7
Morelli A et al,2008	2	0	2	1	5
Morelli A et al,2009	2	0	2	1	5
